# Preparation of Dispersed Platinum Nanoparticles on a Carbon Nanostructured Surface Using Supercritical Fluid Chemical Deposition

**DOI:** 10.3390/ma3031559

**Published:** 2010-03-03

**Authors:** Mineo Hiramatsu, Masaru Hori

**Affiliations:** 1 Department of Electrical and Electronic Engineering, Meijo University, Tempaku, Nagoya 468-8502, Japan; 2 Department of Electrical Engineering and Computer Science, Nagoya University, Chikusa, Nagoya 464-8603, Japan; E-Mail: hori@nuee.nagoya-u.ac.jp (M.H.)

**Keywords:** carbon nanostructures, supercritical fluid, platinum nanoparticles

## Abstract

We have developed a method of forming platinum (Pt) nanoparticles using a metal organic chemical fluid deposition (MOCFD) process employing a supercritical fluid (SCF), and have demonstrated the synthesis of dispersed Pt nanoparticles on the surfaces of carbon nanowalls (CNWs), two-dimensional carbon nanostructures, and carbon nanotubes (CNTs). By using SCF-MOCFD with supercritical carbon dioxide as a solvent of metal-organic compounds, highly dispersed Pt nanoparticles of 2 nm diameter were deposited on the entire surface of CNWs and CNTs. The SCF-MOCFD process proved to be effective for the synthesis of Pt nanoparticles on the entire surface of intricate carbon nanostructures with narrow interspaces.

## 1. Introduction

Graphite-related materials have long been a subject of interest to researchers. Since the first report of carbon nanotubes (CNTs) by Iijima [1], the fabrication of carbon nanostructures has been studied intensively. One-dimensional carbon nanostructures, such as carbon nanotubes and carbon nanofibers, have attracted significant interest for applications such as electrochemical devices, electron field emitters, hydrogen storage materials, and scanning probe microscopy, due to their favorable physical, chemical, and mechanical characteristics [2,3,4].

**Figure 1:**
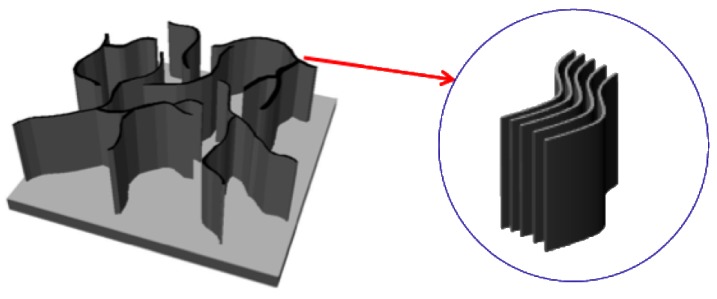
Schematic illustration of carbon nanowalls.

**Figure 2:**
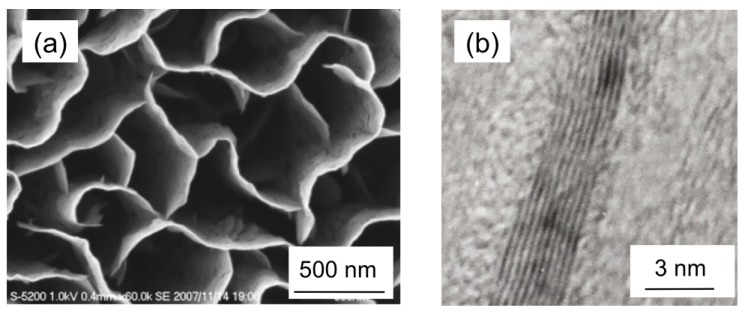
(a) SEM image of typical carbon nanowalls. (b) A high-resolution TEM image of a CNW, showing graphene layers of carbon nanowalls.

Two-dimensional carbon nanostructures have also been grown [5,6,7]. Layered graphene sheets can form two-dimensional carbon nanostructures with edges, called carbon nanowalls (CNWs), carbon nanosheets or carbon nanoflakes. CNWs can be described as graphite sheet nanostructures with edges composed of stacks of planar graphene sheets standing almost vertically on a substrate. The sheets form a wall structure with thicknesses in the range from a few nanometers to few tens of nanometers, and with a high aspect ratio (Figure 1 and Figure 2). Their high aspect ratio and high surface-to-volume ratio are potentially useful as electron field emitters [8,9,10] and as catalyst supports [11]. It is expected that metal nanoparticles supported on the surfaces of carbon nanostructures will improve their electrical properties, and electrocatalyst/carbon nanostructure composites can be applied as electrochemical devices [11].

Among possible supporting materials, carbon black has been widely used as an electrode, wherein platinum (Pt) nanoparticles are dispersed [12,13]. In contrast, CNTs are considered to be a more attractive candidate due to their outstanding characteristics, including high tensile strength, large surface area, high electrical conductivity, and thermal conductivity [14,15,16]. Carbon nanohorns (CNH) and CNWs are also possible candidates for similar applications due to their large surface areas. It has been reported that CNHs can be used as Pt supports for fuel cell applications [17].

It is well known that the specific activity of catalysts is strongly related to their size, dispersion, and compatibility with supporting materials. Highly dispersed catalyst nanoparticles with small size and narrow size distribution supported on carbon nanostructures are ideal for high electrocatalyst activity due to their large surface-to-volume ratio. To support metal nanoparticles on the surface of carbon nanostructures, metal compounds in the form of liquids are generally employed. A few papers have been published on the preparation of Pt nanoparticles on CNT surfaces by the reduction of Pt salt precursors such as H_2_PtCl_6_ in solution [18,19]. On the other hand, it is difficult to treat the entire surface of CNWs with a metal compound in a liquid phase, because of the high surface tension of CNWs due to their high aspect ratio with narrow interspaces. Therefore, there is an urgent need for new methods of metal nanoparticle deposition on carbon nanostructures. One promising method is to employ gas phase deposition, such as sputtering and chemical vapor deposition. However, in gas phase deposition, metal nanoparticles are deposited only around the tops of CNWs and tend to easily clump together, resulting in the formation of larger particles or films on the top of carbon nanostructures.

As an alternative approach to support metal nanoparticles on the surfaces of dense, aligned CNTs and CNWs with narrow interspaces, we demonstrate a method employing metal-organic chemical fluid deposition (MOCFD), where supercritical carbon dioxide (sc-CO_2_) is used as a solvent of metal-organic compounds. The supercritical fluid (SCF) possesses attractive properties of both the gas and the liquid phases. Rapid diffusion and permeation are realized by its gas-like diffusivity and viscosity, while its liquid-like density enables dissolution of a wide range of materials. To produce an SCF phase, the temperature and pressure of the material are required to exceed the critical point. The critical point of sc-CO_2_ exists at 7.38 MPa (72.8 atm) and 31.1 °C. Among SCFs, sc-CO_2_ is particularly attractive since it is environmentally friendly and safe due to its low toxicity, low reactivity and nonflammability.

Cansell and Aymonir have surveyed research on the synthesis of functional nanostructured materials utilizing the specific properties of SCFs over the past five years [20]. Recent advances in the synthesis of CNT composites using SCFs have been reviewed by Liu and Han, with emphasis on metal/CNT, metal-oxide/CNT, and polymer/CNT composites [21]. In the case of Pt deposition, the SCF using sc-CO_2_ was first applied to the preparation of polymer-supported Pt nanoparticles using dimethyl(1,5-cyclooctadiene) platinum(II), (PtMe_2_(cod)) as a precursor [22]. Pt nanoparticles of 5–15 nm size were prepared on the CNT surface by hydrogen reduction of Pt(ll) acetylacetonate, (Pt(acac)_2_) in methanol-modified sc-CO_2_ [23]. Erkey’s group has demonstrated the preparation of Pt nanoparticles on a wide range of materials, including carbon aerogel, carbon black, silica aerogel, alumina, and Nafion [24,25,26,27]. In their method, Pt nanoparticles were prepared by impregnating PtMe_2_(cod) into the substrates from sc-CO_2_ solution for ~6 hours. After depressurization, the impregnated metal-organic compound was reduced to elemental Pt by heat treatment in the presence of nitrogen gas for ~4 hours. It took almost 10 hours to complete the process for the preparation of Pt nanoparticles.

In this work, we have developed a method of formation of Pt nanoparticles using the MOCFD process employing sc-CO_2_ as a solvent of metal-organic compounds, and have demonstrated the synthesis of highly dispersed Pt nanoparticles of 2 nm size without heat treatment after depressurization, on the entire surface of two kinds of carbon nanostructures, CNWs with high aspect ratio and narrow interspaces and aligned CNTs [28,29].

## 2. Experimental

**Figure 3:**
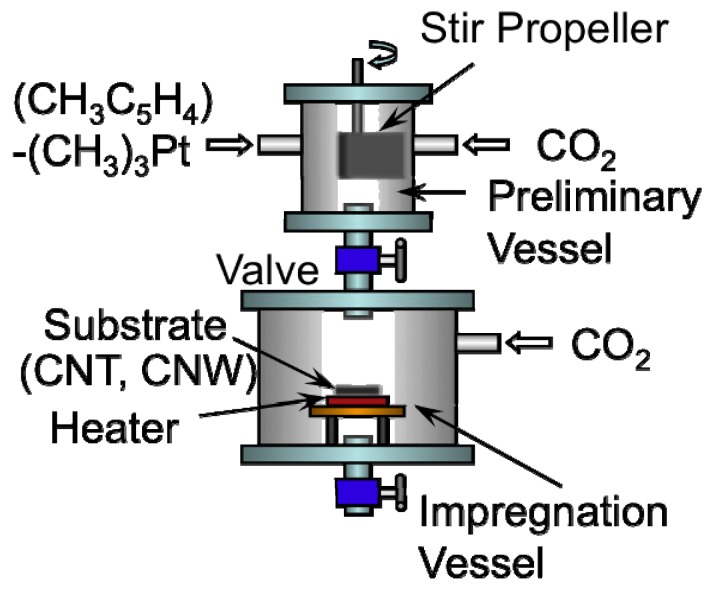
Schematic of the supercritical fluid, metal-organic chemical fluid deposition (SCF-MOCFD) system used in this study [28,29].

Figure 3 shows the SCF-MOCFD system employing sc-CO_2_ used for the deposition of Pt nanoparticles on the surface of CNW and CNT samples [28,29]. The MOCFD process was conducted in two high-pressure stainless steel vessels equipped with a compressor, heating units, and a reservoir for the metal-organic compound. The preliminary vessel contains a screw agitator. The temperature and pressure in each vessel can be set independently, so that two different supercritical conditions employing CO_2_ can be produced in these two vessels. As the precursor, we used (methylcyclopentadienyl) trimethyl platinum ((CH_3_C_5_H_4_)Pt(CH_3_)_3_:MeCpPtMe_3_) dissolved in hexane. The concentration of MeCpPtMe_3_ was 1 wt %, and the quantity of the solution used was 5 mL. In the preliminary vessel, the precursor was stirred with the sc-CO_2_ for about 30 min to realize high diffusion of the metal-organic compound in the sc-CO_2_. In the impregnation vessel, the selective heating of CNW or CNT samples during the MOCFD process facilitated selective metal deposition on the surface of the carbon nanostructures. With help of the needle valve connecting these vessels, it is possible to mix two different fluids and start the metal nanoparticle deposition. In the preliminary vessel, the pressure and temperature of sc-CO_2_ were maintained at 11 MPa and 50 °C, respectively, and MeCpPtMe_3_ was dissolved in the sc-CO_2_. In the impregnation vessel, the pressure and temperature of sc-CO_2_ were maintained at 9 MPa and 70 °C, respectively, and the temperature of CNW or CNT samples was controlled in the range of 70–170 °C. Finally, the solutions were mixed and Pt nanoparticles formation was carried out for 30 min; the vessel was then depressurized slowly in 30 min to atmospheric conditions. After the depressurization, no additional heat treatment was carried out in the present work.

CNW samples were fabricated on a Si substrate by fluorocarbon (C_2_F_6_) plasma enhanced chemical vapor deposition assisted by hydrogen radical injection, comprised of a parallel-plate very high frequency (VHF: 100 MHz), capacitively coupled plasma region, and a hydrogen radical injection source that employs a surface-wave-excited microwave (2.45 GHz) H_2_ plasma [30]. The average height of a typical CNW film sample used in this study was approximately 900 nm, while the thickness of each nanowall was less than 10 nm. Meanwhile, aligned CNT film samples were prepared on the Si substrates using microwave plasma enhanced chemical vapor deposition with a 2.45 GHz, 1.5 kW microwave generator. Details of the synthesis method of the CNT films are described elsewhere [31,32]. A mixture of CH_4_ and H_2_ was used as the source gas. By the controlled preparation of catalytic Co nanoparticles, vertically aligned, single-walled, and double-walled CNT films were fabricated in a controlled manner.

Scanning electron microscopy (SEM) and transmission electron microscopy (TEM) were used to characterize the morphology of the carbon nanostructures and the Pt particle size supported on the surface of the CNWs and CNTs. The presence of Pt was detected by energy dispersive X-ray spectrometry (EDX) and X-ray photoelectron spectroscopy (XPS).

## 3. Results

**Figure 4:**
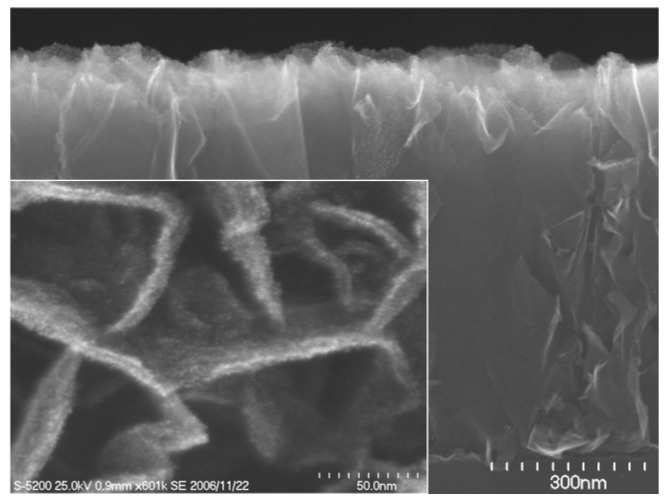
A cross-sectional SEM of a carbon nanowall film after the plating treatment, together with a magnified SEM top view image of the carbon nanowalls in the inset.

**Figure 5:**
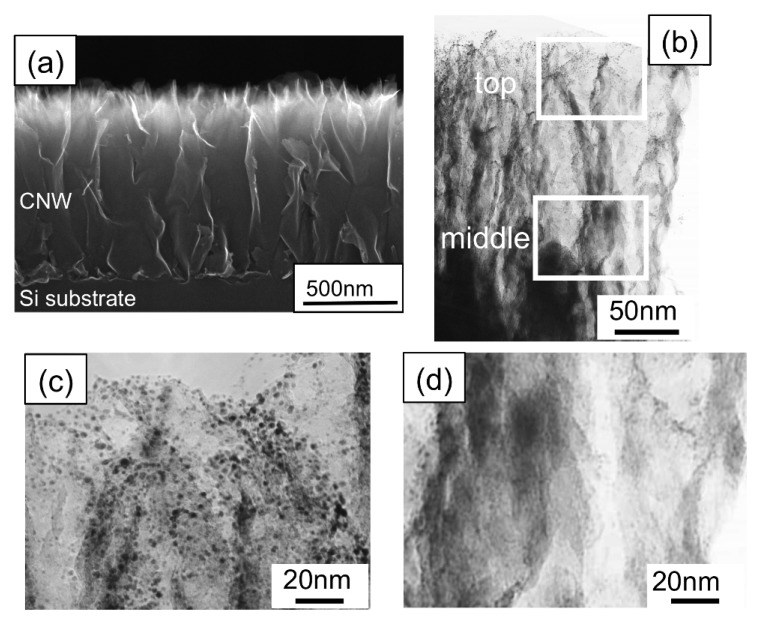
(a) A cross-sectional SEM image of the CNW film, and (b) a low-resolution TEM image of the CNW film after the sputtering treatment. (c) TEM image of the CNW film at the ‘‘top’’ position in the cross-sectional TEM image in Figure 5(b). (d) TEM image of the CNW film at the “middle” position in the cross-sectional TEM image in Figure 5(b) [28].

Figure 4 shows a cross-sectional SEM image of a CNW film after the plating treatment, together with a magnified SEM top view image of the CNWs in the inset. By using the plating method, Pt nanoparticles (5–10 nm) were deposited only on the tops of the CNWs, as shown in the inset in Figure 4. Figure 5(a) and Figure 5(b) show a cross-sectional SEM image and a low-resolution TEM image of CNW film after the sputtering treatment, respectively. Magnified TEM images of CNWs near the top and middle positions are shown in Figure 5(c) and Figure 5(d). In this case, Pt nanoparticles (2–3 nm) were deposited near the top of the CNW film. From the results shown in Figure 4 and Figure 5, it is seen that the preparation of Pt nanoparticles on the entire surface of CNWs could not be realized by deposition methods using the liquid phase or the gas phase.

**Figure 6:**
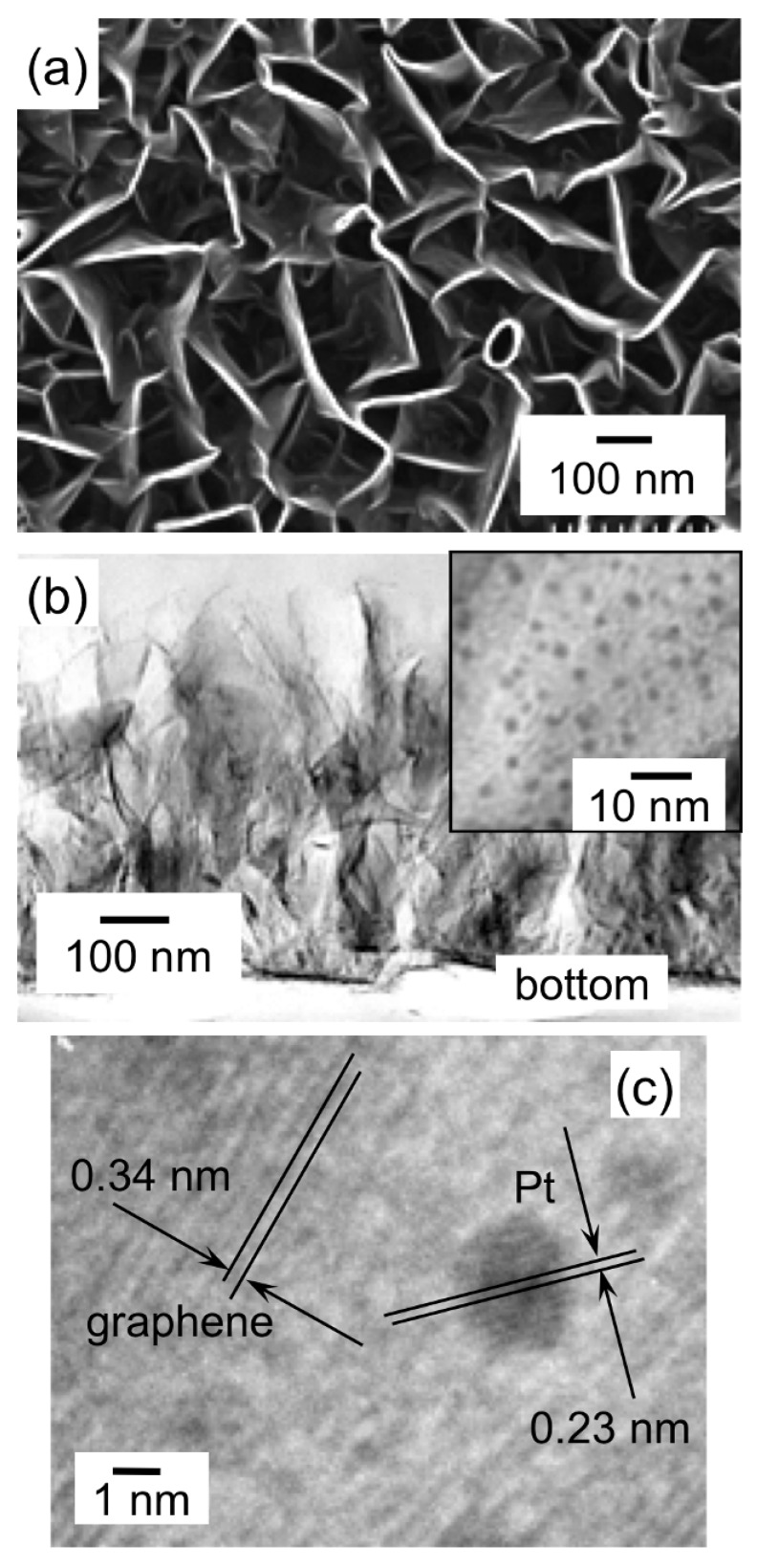
(a) SEM top-view image of a Pt-supported CNW film after SCF-MOCFD treatment at a sample temperature of 150 °C. (b) Low-resolution TEM image of detached CNWs after the SCF-MOCFD treatment. Inset: magnified TEM image of the surface of the CNW supporting Pt nanoparticles. (c) High resolution TEM image showing the d_111_ Pt interplanar distance and graphene layers of the CNW [29].

Figure 6(a) shows an SEM top-view image of a CNW film sample after the SCF-MOCFD treatment at a sample temperature of 150 °C. Compared to the SEM images of typical as-grown CNW films without the SCF treatment (data not shown), no change in the surface morphology was observed. It was confirmed that the unique nanostructure of the CNWs was maintained, even after being exposed to the high-pressure fluid. Figure 6(b) shows a low-resolution TEM image of detached CNWs after the SCF-MOCFD treatment. Nanoparticles are supported on the entire surface of the CNWs. A magnified TEM image of the surface of the CNW after the SCF-MOCFD is shown in the inset in Figure 6(b), indicating the presence of dispersed nanoparticles of approximately 2 nm size on the CNW surface. These nanoparticles have also been identified to be pure platinum by EDX analysis. Figure 6(c) shows a high-resolution TEM image of the surface of the CNW supporting the Pt nanoparticles, and 0.23 nm spaced lattice fringes corresponding to the d_111_ interplanar distance of platinum, well-oriented to the electron beam are observed. The high-resolution TEM image shown in Figure 6(c) also clearly reveals the graphene layers of the CNWs, indicating the graphitized structure of the CNWs. The spacing between neighboring graphene layers was measured to be approximately 0.34 nm.

**Figure 7:**
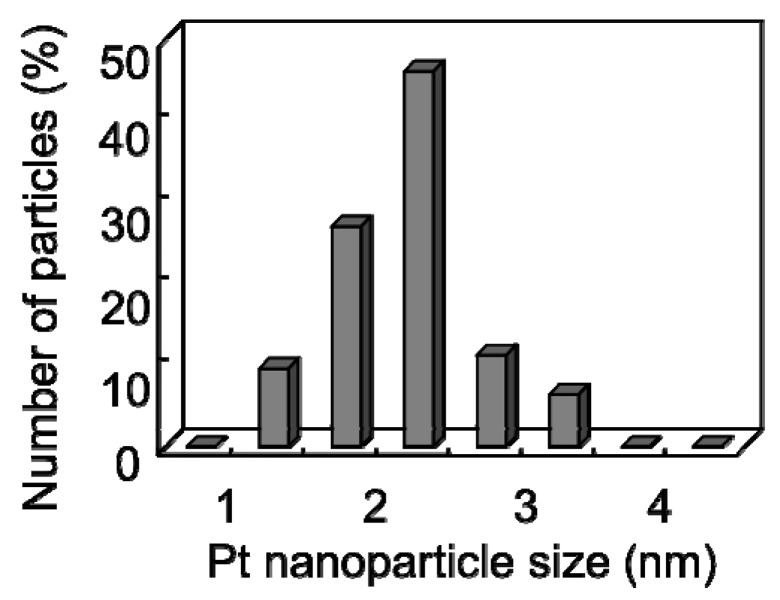
Distribution histogram of Pt nanoparticle size supported on the CNW surface at 150 °C [29].

Figure 7 shows the distribution histogram of the Pt nanoparticle size formed at 150 °C deduced from TEM observations, indicating that the Pt nanoparticles supported on the CNW surface had a small average size and a narrow size distribution centered at approximately 2–2.5 nm.

**Figure 8:**
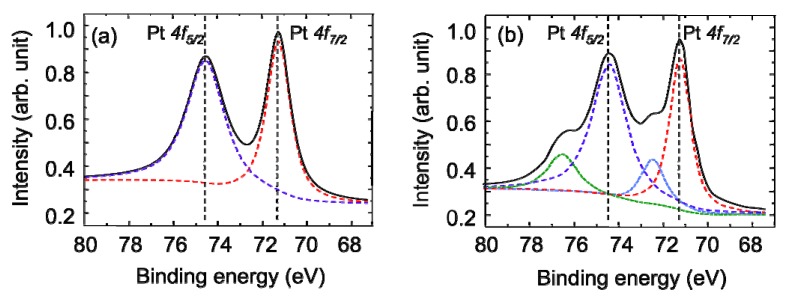
X-ray photoelectron spectroscopy spectra of the Pt-supported CNW film after (a) SCF-MOCFD and (b) plating [29].

*Ex situ* XPS analysis was conducted to gain an insight into the state of the platinum in the supported Pt surface fabricated by the SCF-MOCFD method. Figure 8(a) shows an XPS spectrum of the Pt (4*f*) region of the Pt-supported CNW film after the SCF-MOCFD at a sample temperature of 150 °C. For comparison, Figure 8(b) shows an XPS spectrum of Pt particles deposited on the CNW film after the plating treatment shown in Figure 4. The presence of two prominent sets of Pt (4*f*) peaks, corresponding to the 4*f*_7/2_ and 4*f*_5/2_ orbital states, is further confirmation of platinum being present on the CNW surface. The peak regions in Figure 8(a) can be fitted with two sets of peaks at 71.4 eV (4*f*_7/2_) and 74.6 eV (4*f*_5/2_) [33]. These correspond to platinum in the metallic state, indicating that only pure Pt exists without being oxidized on the surface of the CNWs after the SCF-MOCFD. On the other hand, in the case of the XPS spectrum measured for the Pt particles deposited on the CNW film after the plating treatment shown in Figure 8(b), the peak regions can be fitted with two sets of peaks at 71.4 and 74.6 eV corresponding to platinum in the metallic state, and shoulder peaks at 72.4 and 76.5 eV. These shoulder peaks correspond to platinum in an oxide form [33]. Thus, in the case of deposition by plating, platinum on the surface of the CNW film is present in elemental as well as oxide states.

**Figure 9:**
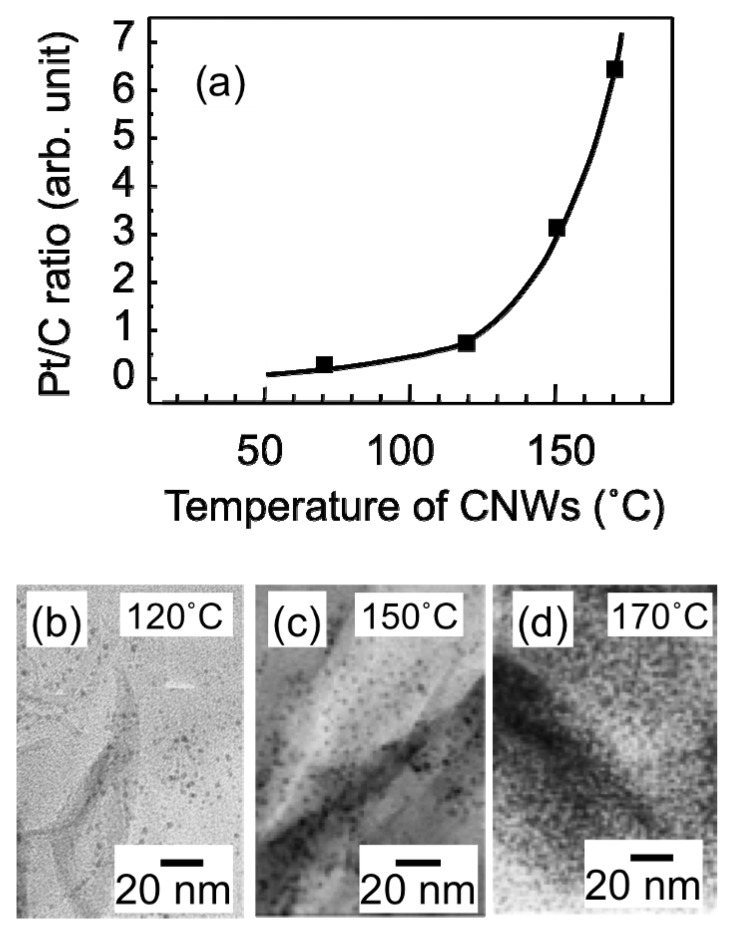
(a) Relative Pt/C ratio of the surface of the CNW film as a function of temperature of the CNW sample during SCF-MOCFD. (b)–(d) High-resolution TEM images of the surface of the CNW supporting Pt nanoparticles after the SCF-MOCFD at sample temperatures of 120, 150, and 170 °C, respectively [29].

The Pt/C ratio of the CNW film surface was obtained from the ratio of the intensities of the XPS C (1*s*) and Pt (4*f*) peaks. Figure 9(a) shows the relative Pt/C ratio of the surface of the CNW film as a function of the temperature of the CNW sample during the SCF-MOCFD process. As the sample temperature during the SCF-MOCFD increased up to 120 °C, the relative Pt/C ratio of the surface of the CNW film increased gradually. By further increasing the sample temperature above 120 °C, the relative Pt/C ratio increased rapidly. Figure 9(b), Figure 9(c), and Figure 9(d) show TEM images of the Pt-supported CNW surface after the SCF-MOCFD at sample temperatures of 120, 150, and 170 °C, respectively. As can be seen from these TEM images, the spatial density of the Pt nanoparticles (particle numbers/area) supported on the CNW surface strongly depended on the sample temperature during the SCF-MOCFD, while the average size of the Pt nanoparticles increased from 1.5 to 3 nm with an increase in the sample temperature from 120 to 170 °C.

**Figure 10:**
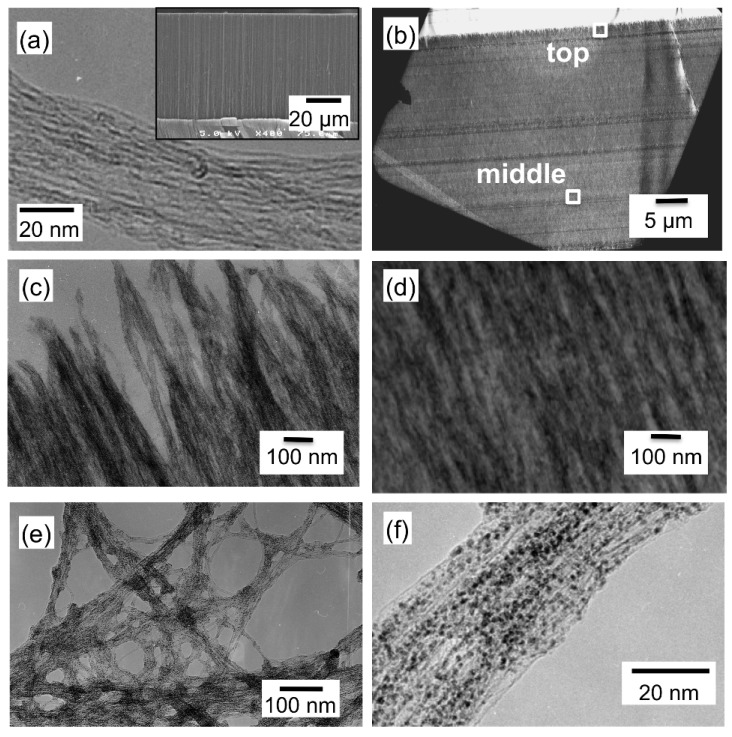
(a) TEM image of CNTs scraped away from the substrate before SCF-MOCFD treatment. Inset: cross-sectional SEM image of a cleaved aligned CNT film grown using microwave plasma-enhanced chemical vapor deposition. (b) Low-resolution cross-sectional TEM image of a Pt-supported CNT film after the SCF-MOCFD treatment at a sample temperature of 150 °C, (c) TEM image of Pt-supported CNT film at the ‘‘top’’ position in the cross-sectional TEM image of Figure 10(b), and (d) TEM image of Pt-supported CNT film at the ‘‘middle’’ position in the cross-sectional TEM image of Figure 10(b). (e) TEM image of CNTs scraped from the substrate after the SCF-MOCFD treatment at a sample temperature of 150 °C. (f) Magnified TEM image of double-walled CNTs scraped from the substrate after the SCF-MOCFD, indicating the presence of dispersed nanoparticles on the CNT surface [29].

A TEM image of CNTs scraped away from the substrate before the SCF-MOCFD treatment is shown in Figure 10(a), together with the cross-sectional SEM image of a cleaved, aligned CNT film as an inset. The TEM image shown in Figure 10(a) reveals that most of the CNTs are double-walled, with an average outer diameter of approximately 4 nm. These vertically aligned, dense CNT films were exposed to the sc-CO_2_ with MeCpPtMe_3_. TEM images of the CNT film after the SCF-CVD are shown in Figure 10(b)–Figure 10(d). Figure 10(b) shows a low-resolution cross-sectional TEM image of the Pt-supported CNT film after the SCF-MOCFD treatment at a sample temperature of 150 °C. The TEM image in Figure 10(c) corresponds to the position indicated as “top” in the cross-sectional TEM image of the CNT film shown in Figure 10(b), while the TEM image in Figure 10(d) corresponds to the “middle” position. The TEM images observed at both positions indicate that the entire surface of the CNTs was covered with platinum. Figure 10(e) shows a TEM image of the CNTs scraped away from the substrate after the SCF-MOCFD treatment at a sample temperature of 150 °C. As a result of the SCF-MOCFD, metal nanoparticles were formed on the entire surface of the CNTs. Figure 10(f) shows a magnified TEM image of double-walled CNTs after the SCF-MOCFD, indicating the presence of dispersed nanoparticles of approximately 2 nm size on the CNT surface.

## 4. Discussion

MeCpPtMe_3_ has been used as a precursor for the formation of Pt particles and films by metal-organic chemical vapor deposition (MOCVD) for years. Thermal decomposition of MeCpPtMe_3_ has been reported to occur at 240 °C, while the initial crystal growth of platinum from the precursors on a highly oriented pyrolytic graphite was observed at 190 °C [34]. In contrast, in our case using the SCF-MOCFD method, the formation of Pt nanoparticles on the surface of carbon nanostructures occurred at relatively low temperatures above 120 °C as shown in Figure 9(a). In an earlier study carried out by Watkins and McCarthy [22], PtMe_2_(cod) was converted to metallic platinum at 140 °C, 26.5 MPa in sc-CO_2_, while the decomposition temperature of PtMe_2_(cod) is 208 °C at atmospheric pressure, which is consistent with our result using the MeCpPtMe_3_ precursor. In the presence of a high-pressure SCF, the metal-organic precursor can be converted to elemental metal at a lower temperature than at atmospheric pressure.

Erkey’s group investigated the particle formation mechanism for a variety of metals using sc-CO_2_ deposition [26,35]. In their method, PtMe_2_(cod) was dissolved in sc-CO_2_ and impregnated into the supporting materials, and after depressurization the impregnated PtMe_2_(cod) molecules were then reduced to metallic Pt nanoparticles by heat treatment or by chemical reduction with hydrogen, resulting in the formation of uniformly dispersed nanoparticles with narrow size distributions. They proposed that the precursor molecules in the sc-CO_2_ phase are adsorbed on the surface, and after depressurization these adsorbed molecules are in turn reduced to elemental platinum and the resulting particles at the surface continue to grow until all the adsorbed precursor molecules are converted to the metal. When the reduction temperature of PtMe_2_(cod) molecules was increased, the resulting particle size increased, due to the increase in mobility of the individual Pt particles, allowing them to coalesce and form larger particles.

In our case, in contrast, the supporting carbon nanostructures were selectively heated in the sc-CO_2_ with Pt precursors during the process. Therefore, at the heated surface of the carbon nanostructures during *in situ* thermal reduction under the SCF environment, decomposition of the adsorbed precursor molecules and growth of the particles would occur. Many defects exist on the surface of CNTs grown by plasma-enhanced chemical vapor deposition. Meanwhile, the CNWs have been reported to consist of nanodomains a few tens of nanometers in size [36], and individual CNWs were found to have many defects [37]. It is suggested that the surface-migrating Pt adatoms produced by the decomposition of MeCpPtMe_3_ precursors merge to form Pt clusters from several Pt atoms preferentially at chemically active sites such as defects and grain boundaries on the surface of the carbon nanostructures, resulting in the nucleation of Pt nanoparticles.

The reaction temperature at the surface would be a significant factor influencing the particle number density and particle size. As pointed out by Erkey’s group [26], when the temperature is increased, both reduction of metal-organic precursors and surface migration of Pt atoms would be enhanced, which may lead to an increase in the particle number density and particle size. As can be seen from the TEM images in Figure 9(b)–Figure 9(d), the average size of Pt nanoparticles increased from 1.5 to 3 nm with an increase in the sample temperature from 120 to 170 °C, while the Pt particle number density increased drastically. The amount of Pt loading supported on the CNW surface has not been measured. Under the constant period of the SCF-MOCFD process, the amount of precursors arriving at the CNW surface is assumed to be always almost the same, in spite of differences in the sample temperatures. Assuming that sufficient amount of precursors arrive at the surface, the Pt nucleation density (nucleation sites/area) would be determined by the rate of reduction of precursor molecules on the surface, which would be enhanced by increasing the surface temperature. Therefore, the amount of Pt loading is considered to increase with an increase in the sample temperature, as can be expected from the TEM images shown in Figure 9(b)–Figure 9(d). Furthermore, with regards to the growth experiment in our study, the processing period including impregnation and reduction was only 30 min, considerably shorter than the typical period of other groups and was not sufficient for aggregation of particles. This could explain the small particle size and size distribution in this study. The large surface area of carbon nanostructures to the relevant precursor amount in the system can also account for the small particle size. If the SCF-MOCFD process is carried out at high surface temperatures for a long period (~hours), the size of the Pt particles would increase and aggregation would likely occur.

## 5. Conclusions

We have developed a new method of deposition using supercritical carbon dioxide to treat the entire surface of carbon nanostructures. We demonstrated the synthesis of dispersed Pt nanoparticles using metal-organic chemical fluid deposition employing the supercritical fluid (SCF-MOCFD). The proposed SCF-MOCFD method proved quite effective for the synthesis of Pt nanoparticles on the entire surface of aligned carbon nanotubes and carbon nanowalls with narrow interspaces. The size of the Pt nanoparticles synthesized at 150 °C was approximately 2 nm.
